# *cytoviewer:* an R/Bioconductor package for interactive visualization and exploration of highly multiplexed imaging data

**DOI:** 10.1186/s12859-023-05546-z

**Published:** 2024-01-03

**Authors:** Lasse Meyer, Nils Eling, Bernd Bodenmiller

**Affiliations:** 1 Department of Quantitative Biomedicine, University of Zurich, Zurich, Switzerland; 2 Institute for Molecular Health Sciences, ETH Zurich, Zurich, Switzerland; 3 Life Science Zurich Graduate School, ETH Zurich/University of Zurich, Zurich, Switzerland

**Keywords:** Visualization, R, Spatial, Single-cell, Multiplexed-imaging, Imaging-mass-cytometry

## Abstract

**Background:**

Highly multiplexed imaging enables single-cell-resolved detection of numerous biological molecules in their spatial tissue context. Interactive visualization of multiplexed imaging data is crucial at any step of data analysis to facilitate quality control and the spatial exploration of single cell features. However, tools for interactive visualization of multiplexed imaging data are not available in the statistical programming language R.

**Results:**

Here, we describe *cytoviewer*, an R/Bioconductor package for interactive visualization and exploration of multi-channel images and segmentation masks. The *cytoviewer* package supports flexible generation of image composites, allows side-by-side visualization of single channels, and facilitates the spatial visualization of single-cell data in the form of segmentation masks. As such, *cytoviewer* improves image and segmentation quality control, the visualization of cell phenotyping results and qualitative validation of hypothesis at any step of data analysis. The package operates on standard data classes of the Bioconductor project and therefore integrates with an extensive framework for single-cell and image analysis. The graphical user interface allows intuitive navigation and little coding experience is required to use the package. We showcase the functionality and biological application of *cytoviewer* by analysis of an imaging mass cytometry dataset acquired from cancer samples.

**Conclusions:**

The *cytoviewer* package offers a rich set of features for highly multiplexed imaging data visualization in R that seamlessly integrates with the workflow for image and single-cell data analysis.

It can be installed from Bioconductor via https://www.bioconductor.org/packages/release/bioc/html/cytoviewer.html. The development version and further instructions can be found on GitHub at https://github.com/BodenmillerGroup/cytoviewer.

**Supplementary Information:**

The online version contains supplementary material available at 10.1186/s12859-023-05546-z.

## Background

Highly multiplexed tissue imaging allows spatial and single-cell-resolved detection of dozens of biological molecules, including proteins and nucleic acids, in situ. These technologies facilitate an in-depth analysis of complex systems and diseases such as the tumor microenvironment [1–3] and type 1 diabetes progression [4]. Imaging-based spatial proteomics methods [5] can be broadly divided into fluorescent cyclic approaches such as cyclic immunofluorescence (t-CyCIF) [6] and co-detection by indexing (CODEX) [7], and one-step mass-tag based approaches that include multiplexed ion beam imaging (MIBI) [8] and imaging mass cytometry (IMC) [9].

To fully leverage the information contained in multiplexed imaging data, the application of computational tools for data analysis is necessary. The main analysis steps, irrespective of the biological question, are (1) image quality control, (2) image pre-processing and segmentation, and (3) single-cell and spatial analysis [10]. Interactive image visualization is essential throughout the entire data analysis workflow and supports key analysis tasks such as segmentation quality control, and hypothesis formulation and verification. With an ever-growing user community [11], this creates the need for easy-to-use image viewers that can visualize all possible results obtained during analysis.

However, popular software, such as histoCAT [12], QuPath [13], and others [14, 15], show little interoperability with programming languages and major frameworks for image and single-cell analysis. The recently developed napari image viewer, which operates in Python, bridges the gap between multiplexed image visualization and data analysis [16], but similar tools that operate in the widely used statistical programming language R have not been developed.

Here, we present *cytoviewer*, an R/Bioconductor package for interactive multi-channel image and segmentation mask visualization in R. The *cytoviewer* package builds on the *cytomapper* R/Bioconductor package [17] and extends its static visualization abilities via an interactive and user-friendly *shiny* application. It provides interactive visualization strategies in a similar fashion as the *iSEE* package [18] offers for single-cell data and can be seamlessly harmonized with any step of the data analysis workflow in R. Users can overlay individual images with segmentation masks, visualize cell-specific metadata, and download generated images. The *cytoviewer* package integrates into the Bioconductor framework [19] for single-cell and image analysis leveraging the image handling and analysis strategies from the *EBImage* Bioconductor package [20] and building on commonly used Bioconductor classes such as *SingleCellExperiment*, *SpatialExperiment* [21, 22], and *CytoImageList* [17]*.* We showcase the functionality and biological application of *cytoviewer* by demonstrating visual exploration of an IMC dataset of cancer patients.

### Implementation

The R/Bioconductor *cytoviewer* package leverages the reactive programming framework of the popular R *shiny* and *shinydashboard* packages [23], is cross-platform compatible, and launches an interactive web application.

**Fig. 1:**
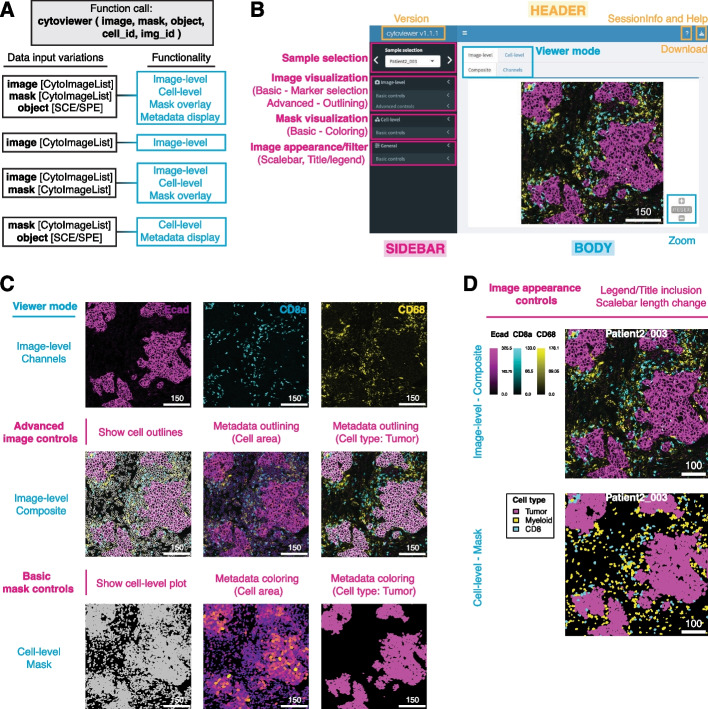
*cytoviewer* interface and functionality. (**A**) The supported functionality (right) of *cytoviewer* depends on the data inputs (left). To match information between the objects, cell (cell_id) and image (img_id) identifiers can be provided. SCE/SPE = *SingleCellExperiment/SpatialExperiment*. (**B**) The graphical user interface of *cytoviewer* is divided into a body, header, and sidebar. The body of *cytoviewer* includes the image viewer, which has three tabs: Composite (Image-level), Channels (Image-level), and Mask (Cell-level). Zooming is supported for Composite and Mask tabs. The package version, R session information, help page, and a drop-down menu for image downloads are located in the header. The sidebar menu has controls for sample selection, image visualization, mask visualization, and general settings. Scale bar: 150 µm (**C**) *cytoviewer* supports different viewing modes. Top: The “channels” tab of image-level visualization displays individual channels. Shown are Ecad (magenta), CD8a (cyan), and CD68 (yellow) marking tumor cells, CD8^+^ T cells, and myeloid cells, respectively. Center: The “composite” tab of image-level visualization visualizes image composites combining multiple channels. These composite images can be overlayed with cell outlines, which can be colored by cell-specific metadata. Shown here are cell outlines colored by cell area (continuous value) and cell type (categorical value; tumor cells in white). Channel color settings are as follows for all markers: Contrast: 2,5; Brightness: 1; Gamma: 1.2. Bottom: The “mask” tab can be used to visualize segmentation masks that can be colored by cell-specific metadata. Shown here are segmentation masks colored by cell area (continuous;) and cell type (categorical; tumor cells in magenta). Scale bars: 150 µm. (**D**) “Image appearance” controls can be used to add legends or titles and to change the scale bar length for image-level (top) and cell level (bottom) visualization. The cell-level mask plot depicts tumor (magenta), myeloid (yellow), and CD8^+^ T cells (cyan). Scale bars: 100 µm

The graphical user interface (GUI) of the *cytoviewer* package can be opened directly from R and the function call takes up to five arguments (Fig. 1A). Images must be provided as a *CytoImageList* object [17] containing one or multiple multi-channel images where each channel represents the pixel intensities of one marker. Segmentation masks in *CytoImageList* format can be added if desired. Segmentation masks are represented as single-channel images containing integer values for cells or zero for background. Furthermore, *SingleCellExperiment* [21] or *SpatialExperiment* [22] class objects can be provided to allow single-cell specific metadata visualization. The full functionality of *cytoviewer* is leveraged when images, segmentation masks, and a metadata object are provided (Fig. 1A). This allows comprehensive image-level and cell-level visualization, enables image overlays with segmentation masks, and cell-specific metadata visualization.

The *cytoviewer* GUI has three main parts: body, sidebar, and header (Fig. 1B). The body of *cytoviewer* features the image viewer. The viewer can switch between image-level visualization, which shows the pixel-level intensities of all selected markers either combined (Composite) or separately (Channels), and cell-level visualization, which displays cell-level information on segmentation masks (Masks) (Additional file 5: Supplementary Notes—S1.1/S1.2). Controls for sample selection, and settings for image and mask visualization are found in the sidebar menu. The header section contains the package version, R session information, a help page, and a drop-down menu for image downloads.

For rapid and publication-quality image downloads, the user specifies a file name, selects the image of interest (Composite, Channels, Mask) and the file format (pdf, png) (Additional file 5: Supplementary Notes—S1.3).

## Results

To demonstrate the functionality and potential biological applications of the *cytoviewer* package, we explored an example IMC dataset from the Integrated iMMUnoprofiling of large adaptive CANcer patient cohort project (immucan.eu) (Additional file 5: Supplementary Notes—S1.4). For IMC, tissue sections are stained with antibodies tagged with isotopically pure rare earth metals, the tissue is laser ablated, and tags are detected by mass spectrometry to produce high-dimensional images [9]. Here, we showcase the different viewing modes of *cytoviewer* by analyses of images from a breast cancer patient (Patient2_003) and outline the rich biological information content for each step (Fig. 1C, Additional file 3: Supplementary Fig. S1).

Image visualization control is split into basic and advanced control modes. Basic controls support the selection of up to six channels with separate color control settings for each (contrast, brightness, gamma, and channel color). In the example shown here, we visualized expression of Ecad, CD8a, and CD68, which are markers for epithelial and tumor cells, CD8^+^ T cells, and macrophages, respectively (Fig. 1B, Fig. 1C—*Top*). This image visualization step supports qualitative cell type identification as well as assessment of signal sensitivity and specificity.

In the advanced image control mode, the user can choose to overlay the displayed images with provided segmentation masks (Fig. 1C—*Center*). Outline color and thickness can be adjusted by the user. This step supports evaluation of cell segmentation quality, which is essential for downstream biological data interpretation. Moreover, the masks can be outlined by cell-specific metadata from the *SingleCellExperiment/SpatialExperiment* object. For categorical and continuous metadata entries, the user can choose between discrete colors and continuous color palettes, respectively. By outlining the masks with the cell area and cell type information (e.g., tumor), correct phenotype assignment can be visually confirmed (e.g., tumor cells are Ecad^+^ and tumor cells have larger areas than other cells).

The user can further decide to display the provided segmentation masks (Fig. 1C—*Bottom*). Coloring of the masks by cell-specific metadata (categorical and continuous) is possible and can be used for visual assessment of spatial tissue features such as tumor cell areas, tumor patch formation, and tumor cell dissemination, among others.

Using image appearance controls, the user can adjust the scale bar length and include legends or image titles. These features can be used for image-level and cell-level visualization and can aid in interpretation of phenotype co-localization such as infiltration of CD8^+^ T cells into the tumor core, which is a clinically relevant tissue feature [24] (Fig. 1D). Furthermore, the image filters section controls pixel-wise interpolation (default) and allows applying Gaussian filters on the image-level (Additional file 4: Supplementary Fig. S2).

## Discussion

The R/Bioconductor *cytoviewer* package provides a rich set of visualization features including (1) visualization of composite and single-channel images, (2) outlining of cells on images, (3) setting cell outline colors based on cellular metadata (e.g., cell phenotype), (4) coloring segmentation masks based on cellular metadata (e.g., cell area) and (5) easy access and download of generated plots for publication, among others. It supports key analysis tasks including cell segmentation quality control, cell phenotype identification and confirmation, and hypothesis formulation and verification. As such, it forms a crucial basis for visually validating a number of biological questions such as: are the identified cell phenotypes correctly labelled and are there biases due to incorrect segmentation? Do certain cell phenotypes interact and are they spatially aggregated? Are certain cell phenotypes enriched on images of a certain condition?

The *cytoviewer* package operates on standard data containers of the Bioconductor project [19] such as *SingleCellExperiment/SpatialExperiment* [21, 22] and *CytoImageList* [17] and can thus be readily implemented into existing R pipelines for image and single-cell analysis. The versatile and easy-to-use graphical user interface of *cytoviewer* further allows accessibility to users with little bioinformatics training and coding experience.

Here, we demonstrated the use of *cytoviewer* by exploring IMC data (Additional file 1, 2). However, data from other multiplexed imaging technologies including t-CyCIF [6], CODEX [7], or MIBI [8], which produce pixel-level intensities and (optionally) segmentation masks, can be interactively visualized with *cytoviewer* as long as the input format is appropriate.

We have integrated *cytoviewer* into our widely adopted data analysis pipeline for multiplexed imaging data [10] (https://bodenmillergroup.github.io/IMCDataAnalysis/) and envision that the package will meet the needs of the fast-growing community of highly multiplexed imaging users [11]. Future developments of the package include an integration with modern imaging file types such as OME-NGFF [25] and spatialData [26] to enable image and single-cell analysis in a programming language agnostic fashion.

## Conclusions

The newly developed R/Bioconductor *cytoviewer* package, written in the statistical programming language R, allows interactive visualization and exploration of multi-channel images and segmentation masks. Alongside the related *cytomappe*r package [17], it builds a well-integrated toolbox for highly multiplexed imaging data visualization that can support all major steps of the image and single-cell analysis workflow in R.

## Availability and requirements

- Project name: *cytoviewer*
- Project home page: https://github.com/BodenmillerGroup/cytoviewer
- Operating system(s): Platform independent
- Programming language: R
- Other requirements: R > = 4.0
- License: None
- Any restrictions to use by non-academics: None

### Supplementary Information

**Additional file 1: Publication analysis code.** Analysis code to reproduce present study.

**Additional file 2: Video demonstration.** A video demonstrating the functionality of *cytoviewer*.

**Additional file 3: Fig. S1.** *cytoviewer* graphical user interface overview. The graphical user interface of *cytoviewer* for the three different viewer modes. Image-level-Composite with basic controls (Top-Left) and advanced controls (Top-Right), Image-level-Channels with basic controls (Middle-Left) and advanced controls (Middle-Right) and Cell-level-Mask with basic controls (Bottom-Left) are shown. For image-level visualization, Ecad (magenta), CD8a (cyan) and CD68 (yellow) marking tumor cells, CD8^+^ T cells and myeloid cells, respectively, are shown and channel color settings are as follows for all markers: Contrast: 2,5; Brightness: 1; Gamma: 1.2. For cell-level visualization, tumor cells (magenta) are highlighted. Note that the Composite and Mask tabs are zoomable. Scale bars: 150 *µ*m.

**Additional file 4: Fig. S2.** *cytoviewer* image filters. Image filter controls are relevant for the image level (here: Composite). Ecad (magenta), CD8a (cyan) and CD68 (yellow) marking tumor cells, CD8^+^ T cells, and myeloid cells, respectively, are shown. Channel color settings are as follows for all markers: Contrast: 2,5; Brightness: 1; Gamma: 1.2. The user can turn on pixel-wise interpolation (Left, default) and off (Center). The white boxes indicate the areas magnified in lower images. Users can also apply a Gaussian filter to the image (Right, sigma: 1.5). Scale bars: 150 *µ*m.

**Additional file 5:** Supplementary Notes.

## Data Availability

All data and materials used for this study are publicly available. Please refer to Additional file 5: Supplementary Notes—S2 Code and data availability for more information, including publication analysis code (Additional file 1) and a video demonstration (Additional file 2).

## Abbreviations

CODEX: Co-detection by indexing
GUI: Graphical user interface
IMC: Imaging mass cytometry
MIBI: Multiplexed ion beam imaging
OME-NGFF: Open microscopy environment-next generation file format
t-CyCIF: Tissue-based cyclic immunofluorescence
