# Supplementary material for: cytoviewer: an R/Bioconductor package for interactive visualization and exploration of highly multiplexed imaging data
Source: BMC Bioinformatics. 2024 Jan 3;25:9. doi: 10.1186/s12859-023-05546-z (PMC10765786; doi:10.1186/s12859-023-05546-z)
Supplement: Supplementary file 1 — Additional file 1: Publication analysis code. Analysis code to reproduce present study. [file 12859_2023_5546_MOESM1_ESM.html]

cytoviewer publication analysis


# cytoviewer publication analysis

Lasse Meyer1,2\*, Nils Eling1,2\*\* and Bernd Bodenmiller1,2

1Department for Quantitative Biomedicine, University of Zurich  
2Institute for Molecular Health Sciences, ETH Zurich

\*lasse.meyer@uzh.ch  
\*\*nils.eling@uzh.ch

#### 9 October 2023

# 1 cytoviewer publication

R markdown file related to:

**cytoviewer: an R/Bioconductor package for interactive visualization
and exploration of highly multiplexed imaging data**

**Lasse Meyer, Nils Eling, Bernd Bodenmiller**

*Software note:* All analysis were performed using Bioconductor 3.17, R
version 4.3.0 (2023-04-21) and cytoviewer version 1.1.1.

# 2 Analysis workflow

## 2.1 Download example dataset

In the present manuscript we use an example imaging mass cytometry (IMC)
cancer dataset to showcase the functionality of `cytoviewer`. This
dataset was generated as part of the Integrated iMMUnoprofiling of large
adaptive CANcer patient cohort project (immucan.eu) and
includes IMC data for 4 cancer patients diagnosed with different tumor
types (head and neck cancer, breast cancer, lung cancer and colorectal
cancer). Of note, the manuscript focuses on visualizing one breast
cancer image (Patient2\_003).

The data input objects were processed with the IMC data analysis
workflow and can
be downloaded from https://zenodo.org/record/8095133.

```
# Download
# Note: Files are saved to the current working directory

options(timeout = 1000) #increases the download timeout

download.file("https://zenodo.org/record/8095133/files/spe.rds",
              destfile = "spe.rds")
download.file("https://zenodo.org/record/8095133/files/images.rds",
              destfile = "images.rds")
download.file("https://zenodo.org/record/8095133/files/masks.rds",
              destfile = "masks.rds")
```

## 2.2 Read data into R

### 2.2.1 Images

The image data was stored as a `CytoImageList` object containing the
spillover corrected multi-channel images (n=14). Each image contains 40
channels and each channel represents the pixel-intensities of one marker
(proteins for IMC). The proteins for this dataset are immuno-oncology
related targets including Ecad, CD8a and CD68, which mark tumor, CD8+ T
cells and myeloid cells, respectively.

```
# Load images
images <- readRDS("images.rds")
images
```

```
## CytoImageList containing 14 image(s)
## names(14): Patient1_001 Patient1_002 Patient1_003 Patient2_001 Patient2_002 Patient2_003 Patient2_004 Patient3_001 Patient3_002 Patient3_003 Patient4_005 Patient4_006 Patient4_007 Patient4_008 
## Each image contains 40 channel(s)
## channelNames(40): MPO HistoneH3 SMA CD16 CD38 HLADR CD27 CD15 CD45RA CD163 B2M CD20 CD68 Ido1 CD3 LAG3 / LAG33 CD11c PD1 PDGFRb CD7 GrzB PDL1 TCF7 CD45RO FOXP3 ICOS CD8a CarbonicAnhydrase CD33 Ki67 VISTA CD40 CD4 CD14 Ecad CD303 CD206 cleavedPARP DNA1 DNA2
```

### 2.2.2 Segmentation masks

The segmentation masks were again stored as a `CytoImageList` object containing
one mask (n=14) for each image. Segmentation masks are defined as one-channel
images containing integer values for cells and zero for background.

```
# Load masks
masks <- readRDS("masks.rds")
masks
```

```
## CytoImageList containing 14 image(s)
## names(14): Patient1_001 Patient1_002 Patient1_003 Patient2_001 Patient2_002 Patient2_003 Patient2_004 Patient3_001 Patient3_002 Patient3_003 Patient4_005 Patient4_006 Patient4_007 Patient4_008 
## Each image contains 1 channel
```

### 2.2.3 Metadata object

The metadata object was stored in `SpatialExperiment` format. It
contained various metadata information in the `colData` slot generated
during the analysis pipeline including patient-level information (such
as indication) and cell-level information (such as cell type and cell
area).

```
# Load spe
spe <- readRDS("spe.rds")
spe
```

```
## class: SpatialExperiment 
## dim: 40 47794 
## metadata(5): color_vectors cluster_codes SOM_codes delta_area
##   filterSpatialContext
## assays(2): counts exprs
## rownames(40): MPO HistoneH3 ... DNA1 DNA2
## rowData names(16): channel name ... marker_class used_for_clustering
## colnames(47794): Patient1_001_1 Patient1_001_2 ... Patient4_008_2844
##   Patient4_008_2845
## colData names(33): sample_id ObjectNumber ... patch_id distToCells
## reducedDimNames(9): UMAP TSNE ... seurat UMAP_seurat
## mainExpName: NULL
## altExpNames(0):
## spatialCoords names(2) : Pos_X Pos_Y
## imgData names(1): sample_id
```

For more details regarding the input objects, please refer to the IMC
data analysis
workflow.

## 2.3 Load cytoviewer

```
# Install cytoviewer 
if (!requireNamespace("BiocManager", quietly = TRUE))
    install.packages("BiocManager")

BiocManager::install("cytoviewer")
```

```
# Load cytoviewer
library(cytoviewer)
```

## 2.4 Run cytoviewer

The `cytoviewer` function takes up to **five arguments** and the
functionality depends on which input objects are provided. For more
information, please refer to the help page found at `?cytoviewer` and
**Figure 1B** of this manuscript.

Here, we showcase the full functionality of `cytoviewer` by providing
the images, segmentation masks and metadata object that were described
above.

To match information between the `CytoImageList` objects (images, masks)
and the `SpatialExperiment` object (spe), two additional spots are
specified:

*img\_id*: a single character indicating the `colData` (of the
`SpatialExperiment` object) and `elementMetadata` (of the
`CytoImageList` object) entry that contains the image identifiers. These
image ids have to match between the `SpatialExperiment` object and the
`CytoImageList` objects.

*cell\_id*: a single character indicating the `colData` entry that
contains the cell identifiers. These should be integer values
corresponding to pixel-values in the segmentation masks.

```
# Create shiny app with cytoviewer
app <- cytoviewer(image = images, 
                  mask = masks, 
                  object = spe, 
                  img_id = "sample_id", 
                  cell_id = "ObjectNumber")

# Launch app in your web browser
if (interactive()) {
  shiny::runApp(app, launch.browser = TRUE)
  }
```

### 2.4.1 Additional information

For more detailed information on the functionality of `cytoviewer`,
please refer to the **package vignette** of *cytoviewer*.

### 2.4.2 Publication figure settings

To re-create the figures of the present manuscript, please follow the
instructions below **in order**:

### 2.4.3 Figure 1

**Figure 1A (Step 1)**:

Viewer mode: Image-level - Composite

Sample selection: Select *Patient2\_003*

Image-level - Basic controls:

Select *Ecad* (magenta), *CD8a* (cyan) and *CD68* (yellow) with
following color control settings: Contrast: 2,5; Brightness: 1; Gamma:
1.2

**Figure 1C**:

**Top (Step 2)**:

Change Viewer mode: Image-level - Channels

**Middle/Left (Step 3)**:

Change Viewer mode: Image-level - Composite

Image-level - Advanced controls: Show cell outlines - Outline thickness
control: Select thick

**Middle/Center (Step 4)**:

Image-level - Advanced controls: Show cell outlines - Select *area* for
*Outline by*` - Choose *plasma* for outline color control

**Middle/Right (Step 5)**:

Image-level - Advanced controls: Show cell outlines - Select *celltype*
for *Outline by* - Choose *white* for outline color control

**Bottom/Left (Step 6)**:

Change Viewer mode: Cell-level - Mask

Cell-level - Basic controls: Show cell-level plot

**Bottom/Center (Step 7)**:

Cell-level - Basic controls: Show cell-level plot - Select *area* for
*Color by* - Choose *plasma* for color control

**Bottom/Right (Step 8)**:

Cell-level - Basic controls: Show cell-level plot - Select *celltype*
for *Color by* - Choose *magenta* for tumor color control and *black*
for missing color

**Figure 1D**:

**Top (Step 9)**:

Go back to the settings at *Step 1*.

General - Basic controls - Image appearance: Change *Scale bar length*
to 100 and tick *Show Legend* and *Show Title*

**Bottom (Step 10)**:

Go back to the settings at *Step 8*.

Cell-level - Basic controls: Show cell-level plot - Select *celltype*
for *Color by* - Choose *Tumor*, *CD8* and *Myeloid* for *Select color
by* - Choose *magenta*, *cyan*, *yellow*, *black* for tumor, CD8,
myeloid and missing color color control

General - Basic controls - Image appearance: Change *Scale bar length*
to 100 and tick *Show Legend* and *Show Title*

### 2.4.4 Supplemental Figure 2

**Left (Step 11)**:

Go back to the settings at *Step 1*.

**Center (Step 12)**:

General - Basic controls - Image filters: Un-tick *Pixel-wise
interpolation*

**Right (Step 13)**:

General - Basic controls - Image filters: Tick *Gaussian filter* and set
it to 1.5

### 2.4.5 Example cytomapper function usage

The R/Bioconductor `cytoviewer` package builds on top of the
R/Bioconductor `cytomapper` package
(https://bioconductor.org/packages/release/bioc/html/cytomapper.html)
and utilizes its functions and data containers.

Therefore, we can re-create images that were interactively generated
with `cytoviewer` using `cytomapper`. For more information refer to
**Supplementary Note S1.1**.

```
# Install cytomapper
if (!requireNamespace("BiocManager", quietly = TRUE))
    install.packages("BiocManager")

BiocManager::install("cytomapper")
```

```
# Load cytomapper 
library(cytomapper)
```

Here, we showcase one example for **Figure 1A** of the manuscript using
the `plotPixels` function from `cytomapper`.

```
# Re-create Figure 1A - Composite
plotPixels(image = images["Patient2_003"],
           colour_by = c("Ecad", "CD8a", "CD68"),
           colour = list(Ecad = c("black", "magenta"), 
                         CD8a = c("black","cyan"),
                         CD68 = c("black", "yellow")),
           bcg = list(Ecad = c(1, 2.5, 1.2), 
                      CD8a = c(1, 2.5, 1.2),
                      CD68 = c(1, 2.5, 1.2)),
           legend = NULL,
           image_title = NULL,
           scale_bar = list(length = 150)
           )
```

```
sessionInfo()
```

```
## R version 4.3.0 (2023-04-21)
## Platform: aarch64-apple-darwin20 (64-bit)
## Running under: macOS Monterey 12.2.1
## 
## Matrix products: default
## BLAS:   /Library/Frameworks/R.framework/Versions/4.3-arm64/Resources/lib/libRblas.0.dylib 
## LAPACK: /Library/Frameworks/R.framework/Versions/4.3-arm64/Resources/lib/libRlapack.dylib;  LAPACK version 3.11.0
## 
## locale:
## [1] en_US.UTF-8/en_US.UTF-8/en_US.UTF-8/C/en_US.UTF-8/en_US.UTF-8
## 
## time zone: Europe/Zurich
## tzcode source: internal
## 
## attached base packages:
## [1] stats4    stats     graphics  grDevices utils     datasets  methods  
## [8] base     
## 
## other attached packages:
##  [1] cytoviewer_1.1.1            cytomapper_1.12.0          
##  [3] SingleCellExperiment_1.22.0 SummarizedExperiment_1.30.1
##  [5] Biobase_2.60.0              GenomicRanges_1.52.0       
##  [7] GenomeInfoDb_1.36.0         IRanges_2.34.0             
##  [9] S4Vectors_0.38.1            BiocGenerics_0.46.0        
## [11] MatrixGenerics_1.12.0       matrixStats_0.63.0         
## [13] EBImage_4.42.0              BiocStyle_2.28.0           
## 
## loaded via a namespace (and not attached):
##   [1] bitops_1.0-7              gridExtra_2.3            
##   [3] rlang_1.1.1               magrittr_2.0.3           
##   [5] svgPanZoom_0.3.4          shinydashboard_0.7.2     
##   [7] compiler_4.3.0            DelayedMatrixStats_1.22.0
##   [9] systemfonts_1.0.4         png_0.1-8                
##  [11] fftwtools_0.9-11          vctrs_0.6.2              
##  [13] pkgconfig_2.0.3           SpatialExperiment_1.10.0 
##  [15] crayon_1.5.2              fastmap_1.1.1            
##  [17] magick_2.7.4              XVector_0.40.0           
##  [19] ellipsis_0.3.2            fontawesome_0.5.1        
##  [21] scuttle_1.9.4             utf8_1.2.3               
##  [23] promises_1.2.0.1          rmarkdown_2.21           
##  [25] ggbeeswarm_0.7.2          xfun_0.39                
##  [27] zlibbioc_1.46.0           cachem_1.0.8             
##  [29] beachmat_2.16.0           jsonlite_1.8.4           
##  [31] highr_0.10                later_1.3.1              
##  [33] rhdf5filters_1.12.1       DelayedArray_0.26.7      
##  [35] Rhdf5lib_1.22.0           BiocParallel_1.34.1      
##  [37] terra_1.7-29              jpeg_0.1-10              
##  [39] tiff_0.1-11               parallel_4.3.0           
##  [41] R6_2.5.1                  bslib_0.4.2              
##  [43] RColorBrewer_1.1-3        limma_3.56.1             
##  [45] jquerylib_0.1.4           Rcpp_1.0.10              
##  [47] bookdown_0.34             knitr_1.42               
##  [49] R.utils_2.12.2            httpuv_1.6.11            
##  [51] Matrix_1.5-4              nnls_1.4                 
##  [53] tidyselect_1.2.0          viridis_0.6.3            
##  [55] rstudioapi_0.14           abind_1.4-5              
##  [57] yaml_2.3.7                miniUI_0.1.1.1           
##  [59] codetools_0.2-19          lattice_0.21-8           
##  [61] tibble_3.2.1              shiny_1.7.4              
##  [63] evaluate_0.21             archive_1.1.5            
##  [65] shinycssloaders_1.0.0     pillar_1.9.0             
##  [67] BiocManager_1.30.20       generics_0.1.3           
##  [69] sp_1.6-0                  RCurl_1.98-1.12          
##  [71] ggplot2_3.4.2             sparseMatrixStats_1.12.0 
##  [73] munsell_0.5.0             scales_1.2.1             
##  [75] xtable_1.8-4              glue_1.6.2               
##  [77] tools_4.3.0               colourpicker_1.2.0       
##  [79] locfit_1.5-9.7            rhdf5_2.44.0             
##  [81] grid_4.3.0                DropletUtils_1.20.0      
##  [83] edgeR_3.42.2              colorspace_2.1-0         
##  [85] GenomeInfoDbData_1.2.10   raster_3.6-20            
##  [87] beeswarm_0.4.0            HDF5Array_1.28.1         
##  [89] vipor_0.4.5               cli_3.6.1                
##  [91] fansi_1.0.4               viridisLite_0.4.2        
##  [93] S4Arrays_1.0.4            svglite_2.1.1            
##  [95] dplyr_1.1.2               gtable_0.3.3             
##  [97] R.methodsS3_1.8.2         sass_0.4.6               
##  [99] digest_0.6.31             dqrng_0.3.0              
## [101] rjson_0.2.21              htmlwidgets_1.6.2        
## [103] memoise_2.0.1             htmltools_0.5.5          
## [105] R.oo_1.25.0               lifecycle_1.0.3          
## [107] mime_0.12
```
