# Supplementary figures and images for: cytoviewer: an R/Bioconductor package for interactive visualization and exploration of highly multiplexed imaging data
Source: BMC Bioinformatics. 2024 Jan 3;25:9. doi: 10.1186/s12859-023-05546-z (PMC10765786; doi:10.1186/s12859-023-05546-z)

## Viewer mode

## Image-level - Composite

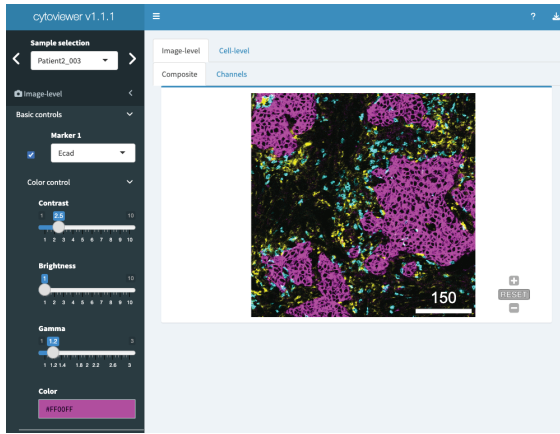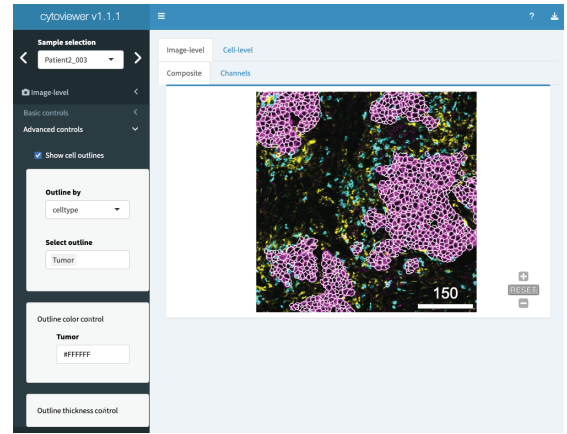

## Image-level - Channels

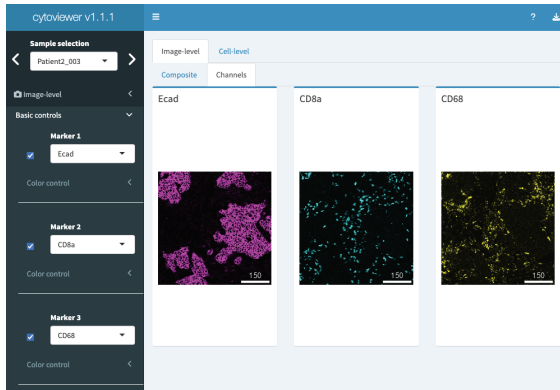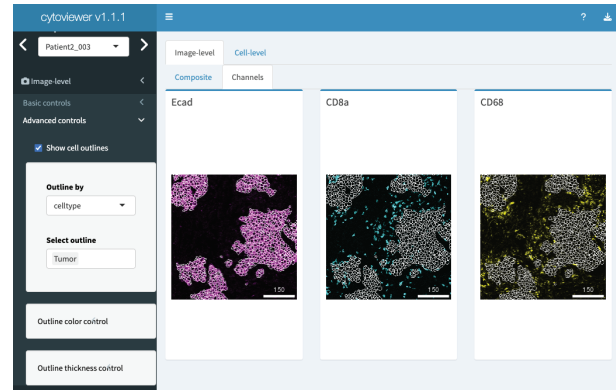

## Cell-level - Mask

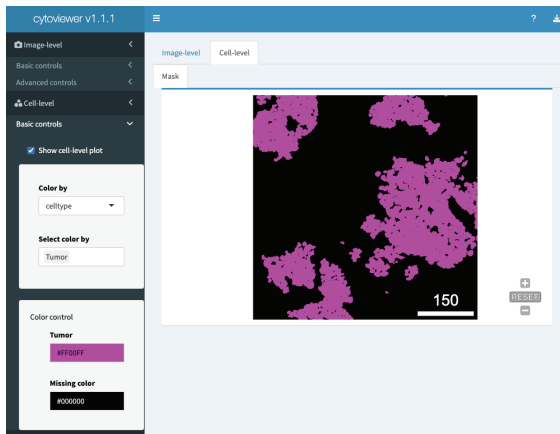

Supplement: Supplementary file 3 — Additional file 3: Fig. S1. cytoviewer graphical user interface overview. The graphical user interface of cytoviewer for the three different viewer modes. Image-level-Composite with basic controls (Top-Left) and advanced controls (Top-Right), Image-level-Channels with basic controls (Middle-Left) and advanced controls (Middle-Right) and Cell-level-Mask with basic controls (Bottom-Left) are shown. For image-level visualization, Ecad (magenta), CD8a (cyan) and CD68 (yellow) marking tumor cells, CD8+ T cells and myeloid cells, respectively, are shown and channel color settings are as follows for all markers: Contrast: 2,5; Brightness: 1; Gamma: 1.2. For cell-level visualization, tumor cells (magenta) are highlighted. Note that the Composite and Mask tabs are zoomable. Scale bars: 150 µm. [file 12859_2023_5546_MOESM3_ESM.pdf]

Image filters  
controls

Default

No pixel-wise interpolation

Gaussian filter (Sigma: 1.5)

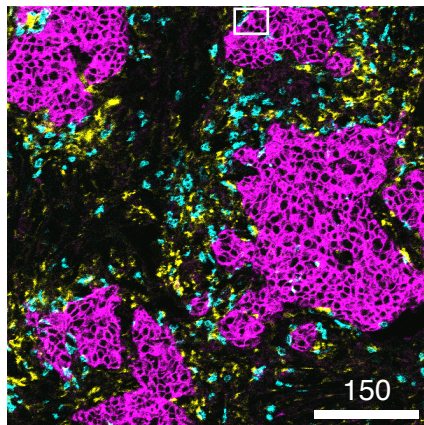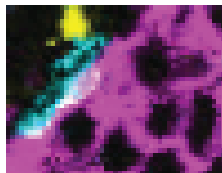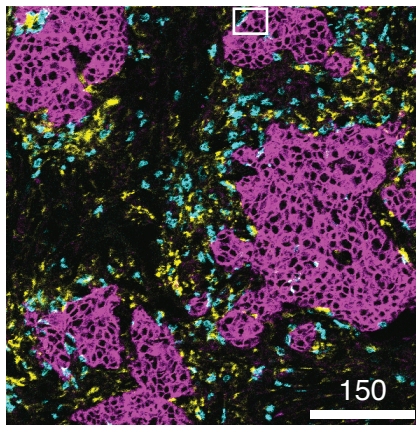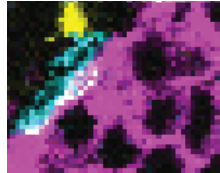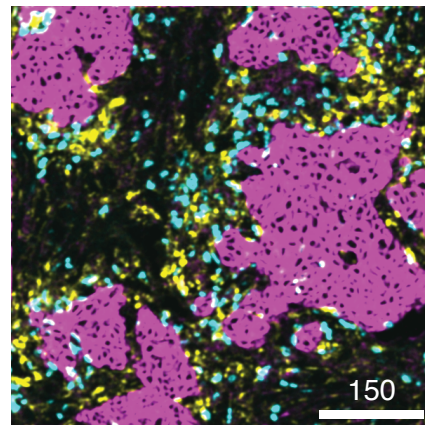

Supplement: Supplementary file 4 — Additional file 4: Fig. S2. cytoviewer image filters. Image filter controls are relevant for the image level (here: Composite). Ecad (magenta), CD8a (cyan) and CD68 (yellow) marking tumor cells, CD8+ T cells, and myeloid cells, respectively, are shown. Channel color settings are as follows for all markers: Contrast: 2,5; Brightness: 1; Gamma: 1.2. The user can turn on pixel-wise interpolation (Left, default) and off (Center). The white boxes indicate the areas magnified in lower images. Users can also apply a Gaussian filter to the image (Right, sigma: 1.5). Scale bars: 150 µm. [file 12859_2023_5546_MOESM4_ESM.pdf]
