# Supplementary material for: cytoviewer: an R/Bioconductor package for interactive visualization and exploration of highly multiplexed imaging data
Source: BMC Bioinformatics. 2024 Jan 3;25:9. doi: 10.1186/s12859-023-05546-z (PMC10765786; doi:10.1186/s12859-023-05546-z)
Supplement: Supplementary file 5 — Additional file 5: Supplementary Notes. [file 12859_2023_5546_MOESM5_ESM.docx]

**Supplementary Notes related to:**

*cytoviewer:* an R/Bioconductor package for interactive visualization and exploration of highly multiplexed imaging data

Lasse Meyer, Nils Eling, and Bernd Bodenmiller

Table of Content:

S1 Supplementary Notes:

S1.1 Function usage of different viewing modes

S1.2 Notes on scalability

S1.3 Download options

S1.4 The example IMC cancer dataset

S2 Code and data availability

S3 Supplementary Figures:

Supplementary Figure S1 - *cytoviewer* graphical user interface overview

Supplementary Figure S2 - *cytoviewer* image filters

S4 References

**S1 Supplementary Notes**

**S1.1 Function usage and defaults for different viewing modes**

The *cytoviewer* package builds on the R/Bioconductor *cytomapper* package (1) and utilizes its functions and data containers. Image-level visualization of the *cytoviewer* package is based on the *plotPixels* function of the R/Bioconductor *cytomapper* package (1). For more information, please refer to the help page found at ***?cytomapper::plotPixels*** and to the *cytomapper* **package vignette.**

In *cytoviewer*, basic controls for image-level visualization allow selection of up to six markers/channels with the following default colors: magenta, cyan, yellow, red, green, and blue. Colors are scaled between the minimum and maximum pixel intensities across the displayed image. Therefore, when selecting images of different samples, the range of pixel intensities can change. Advanced image controls support the overlay of images with segmentation masks (default color: white). When outlining the masks by cell-specific metadata, categorical and continuous metadata entries are colored using discrete colors (from the *RColorBrewer::brewer_pal()* function) and continuous color palettes (viridis, inferno, plasma from the *viridis* package). The “composite” tab of image-level visualization has zoom-controls.

Cell-level visualization of the *cytoviewer* package is based on the *plotCells* function of the R/Bioconductor *cytomapper* package (1). For more information, please refer to the help page found at ***?cytomapper::plotCells*** and to the *cytomapper* **package vignette.** Basic controls for cell-level visualization allow display (default color: gray) and coloring of masks by cell-specific metadata. Akin to image-level visualization, categorical and continuous metadata entries are colored using discrete colors (from the *RColorBrewer::brewer_pal()* function) and continuous color palettes (viridis, inferno, plasma from the *viridis* package). The “masks” tab of cell-level visualization has zoom-controls.

**S1.2 Notes on scalability**

In *cytoviewer*, the rendering time for image-level visualization increases with the number of selected channels and if cell outlines are plotted (Advanced controls). This is specifically noticeable when visualizing individual channels in the “Channels” tab. Moreover, the rendering time increases with the size of the images (i.e., number of pixels in x,y). The displayed images contain 600x600 pixels and composite image rendering occurs instantaneously.

**S1.3 Download options**

Image download controls are part of the header section as a drop-down menu. The user can specify a file name and select the image of interest (Composite, Channels, Mask) and the file format (pdf, png). When the download button is clicked, a pop-up window will appear where the user can specify the download location on the local machine. Individual images from the channels viewing mode are downloaded as a *.zip* file.

**S1.4 The example IMC cancer dataset**

The Imaging Mass Cytometry (IMC) dataset used for *cytoviewer* demonstration was generated as part of the Integrated iMMUnoprofiling of large adaptive CANcer patient cohort project ([immucan.eu](https://uzh.sharepoint.com/sites/cytoviewermanuscript/Shared%20Documents/General/immucan.eu)) with the Hyperion instrument (<https://www.standardbio.com/products/instruments/hyperion>). The dataset includes images of samples from four cancer patients diagnosed with different tumor types (head and neck cancer, breast cancer, lung cancer and colorectal cancer). For this demonstration, images from a breast cancer patient (Patient2_003) were used.

The data input objects were processed with the IMC data analysis workflow (<https://bodenmillergroup.github.io/IMCDataAnalysis/>) using functionality from the *steinbock* framework (2) and the *imcRtools* package (<https://github.com/BodenmillerGroup/imcRtools>) among others. Data were downloaded from <https://zenodo.org/record/8095133/>. The image data were stored as a *CytoImageList* object containing the spillover corrected multi-channel images, and the mask *CytoImageList* object was used to store single-channel segmentation masks. The single-cell data were stored in *SpatialExperiment* format (3). Metadata information generated during the analysis was stored in the *colData* slot. For more details, please refer to <https://bodenmillergroup.github.io/IMCDataAnalysis/>.

**S2 Code and data availability**

All analyses were performed using Bioconductor 3.17, R version 4.3.0 and *cytoviewer* version 1.1.1.

Analysis code to reproduce present study is available:

**Additional File 1 - cytoviewer_publication_analysis.html**

A video demonstrating the functionality of *cytoviewer* is available:

**Additional File 2 - cytoviewer_demo.mp4**

The *cytoviewer* package can be installed from Bioconductor:

<https://www.bioconductor.org/packages/release/bioc/html/cytoviewer.html>

The development version of *cytoviewer* can be found on GitHub:

<https://github.com/BodenmillerGroup/cytoviewer>

A static website with further instructions on package usage and functionality can be found at:

<https://bodenmillergroup.github.io/cytoviewer/>

The example IMC dataset used for the present publication is available at: <https://zenodo.org/record/8095133/>

**S3 Supplementary Figures:**

**Supplementary Figure S1: *cytoviewer* graphical user interface overview.**

The graphical user interface of *cytoviewer* for the three different viewer modes. Image-level-Composite with basic controls (top-left) and advanced controls (top-right), Image-level-Channels with basic controls (middle-left) and advanced controls (middle-right) and Cell-level-Mask with basic controls (bottom-left) are shown. For image-level visualization, Ecad (magenta), CD8a (cyan) and CD68 (yellow) marking tumor cells, CD8+ T cells and myeloid cells, respectively, are shown and channel color settings are as follows for all markers: Contrast: 2,5; Brightness: 1; Gamma: 1.2. For cell-level visualization, tumor cells (magenta) are highlighted. Note that the Composite and Mask tabs are zoomable. Scale bars: 150 µm.

**
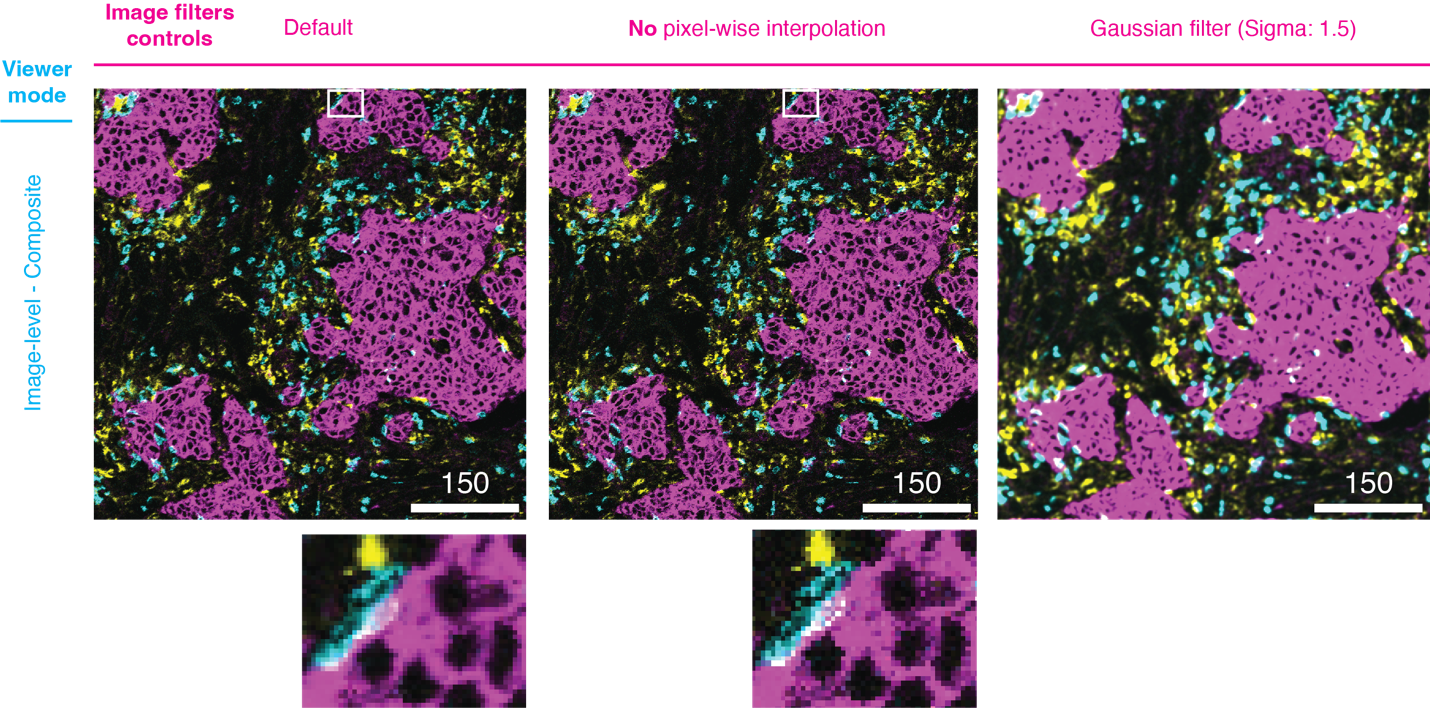
Supplementary Figure S2: *cytoviewer* image filters.** Image filter controls are relevant for the image level (here: Composite). Ecad (magenta), CD8a (cyan) and CD68 (yellow) marking tumor cells, CD8^+^ T cells, and myeloid cells, respectively, are shown. Channel color settings are as follows for all markers: Contrast: 2,5; Brightness: 1; Gamma: 1.2. The user can turn on pixel-wise interpolation (left, default) and off (center). The white boxes
indicate the areas magnified in lower images. Users can also apply a Gaussian filter to the image (right, sigma: 1.5). Scale bars: 150 µm.

**S4 References**

1. Eling N, Damond N, Hoch T, Bodenmiller B. cytomapper: an R/Bioconductor package for visualization of highly multiplexed imaging data. Bioinformatics [Internet]. 2020 Dec 15;36(24):5706–8. Available from: https://doi.org/10.1093/bioinformatics/btaa1061

2. Windhager J, Bodenmiller B, Eling N. An end-to-end workflow for multiplexed image processing and analysis. bioRxiv [Internet]. 2021 Jan 1;2021.11.12.468357. Available from: http://biorxiv.org/content/early/2021/11/13/2021.11.12.468357.abstract

3. Righelli D, Weber LM, Crowell HL, Pardo B, Collado-Torres L, Ghazanfar S, et al. SpatialExperiment: infrastructure for spatially-resolved transcriptomics data in R using Bioconductor. Bioinformatics [Internet]. 2022 May 26;38(11):3128–31. Available from: https://doi.org/10.1093/bioinformatics/btac299
